# Content and Yield of L-DOPA and Bioactive Compounds of Broad Bean Plants: Antioxidant and Anti-Inflammatory Activity In Vitro

**DOI:** 10.3390/plants12233918

**Published:** 2023-11-21

**Authors:** Paula Beatriz Fuentes-Herrera, Braulio Edgar Herrera-Cabrera, Alma Leticia Martínez-Ayala, Alejandro Zamilpa, Adriana Delgado-Alvarado

**Affiliations:** 1 Campus Puebla, Colegio de Posgraduados, 72760 Puebla, Mexico; pau852012@hotmail.com (P.B.F.-H.); behc@colpos.mx (B.E.H.-C.); 2 Centro de Desarrollo de Productos Bióticos, Instituto Politécnico Nacional, 62730 Morelos, Mexico; alayala@ipn.mx; 3 Centro de Investigación Biomédica del Sur (CIBIS), Instituto Mexicano del Seguro Social (IMSS), 62790 Morelos, Mexico; azamilpa_2000@yahoo.com.mx

**Keywords:** field, Parkinson’s disease, flavonoids, isoorientins, yield, rutin, *Vicia faba* L.

## Abstract

The broad bean plant contains L-DOPA, a compound that is essential for patients with Parkinson’s disease. However, little has been reported on other broad bean compounds that have beneficial effects on health. The objective was to evaluate plants of four Mexican broad bean varieties to determine the content and yield of total phenolic compounds (TPC), total flavonoids (TF), and L-DOPA, as well as to analyze the flavonoid profile and antioxidant (AA) and anti-inflammatory (AANTI) activity in vitro. Broad bean seeds were sown in the field and plants were harvested 20 days after emergence. The analyses were performed with visible UV spectrophotometry and HPLC. The variety José María produced the highest yield of TPC (9.30 g m^−2^), TF (8.08 g m^−2^), and L-DOPA (5.64 g m^−2^) per unit of area. The highest yields per plant were obtained with the Rojita variety: TPC (0.25 g plant^−1^), TF (0.21 g plant^−1^), and L-DOPA (0.17 g plant^−1^). This variety also had the highest antioxidant (IC_50_ = 87.68 µg mL^−1^) and anti-inflammatory (IC_50_ = 74.40 mg mL^−1^) activity, which was attributed to the L-DOPA compounds and to rutin and isoorientins, respectively. The flavonoid profile revealed the presence of rutin and isoorientins, which had not been previously detected in the broad bean plant.

## 1. Introduction

*Vicia faba* L. belongs to the Fabaceae family [1]. Only the fruit of this legume is used for human or animal consumption because of its nutritional and functional properties [2]. The plant contains important quantities of phenolic compounds that can be extracted [3], such as L-3,4-dihydroxyphenylalanine (L-DOPA) that is located in different plant structures or organs [4,5]. Particularly, in seedlings 15 days after germination, a high concentration of L-DOPA is accumulated but tends to decrease in different organs in the plant’s physiologically mature stage [5]. Moreover, the seedlings can contain up to twenty times more L-DOPA than the fruits [6]. L-DOPA is prescribed in the treatment of patients with Parkinson’s disease (PD) since it helps to restore dopamine in the brain. The disease is related to a decrease in the production of the neurotransmitter dopamine. This alteration is caused by the degeneration of dopaminergic neurons in the substantia nigra, causing symptoms of bradykinesia, rigidity, tremor, and postural and gait disturbances [7]. L-DOPA is administered to PD patients through synthetic medicines such as Sinemet^®^, Atamet^®^, Parcopa^®^, and Stalevo^®^ [8]. Nevertheless, it has been proven in patients with PD that consuming cooked broad beans (250 g) significantly improves motor ability, similar to improvement by ingesting synthetic levodopa (125 mg) plus carbidopa (12.5 mg) [9]. The consumption of fresh broad bean seedlings (40 g provides approximately 120–130 mg L-DOPA) has also been shown to have a beneficial effect: the concentration of L-DOPA increased in the blood plasma of patients who, in turn, exhibited better motor characteristics, similar to that when the drug is administered [6].

*V. faba* could be considered a medicinal plant because of its beneficial contribution to PD patients. Medicinal plants play an important role in health. Although the properties of several plants have not been studied, they may be decisive in the treatment of present-day or future diseases [10]. The use of medicinal plants has been growing. Compared with synthetic medicines, they are affordable and accessible for people with low incomes, and their intake does not have the secondary effects that conventional medicines can cause [11]. Recently, the World Health Organization (WHO) reported that the most effective medicine for PD, levodopa with carbidopa, is not accessible, affordable, or available everywhere, especially in countries with low or medium incomes [12]. The *V. faba* plant, because it is a natural source of L-DOPA, should be cultivated to produce the metabolite. For example, the stems and leaves of plants 75 days after sowing (DAS) have yields of L-DOPA of up to 55.2 kg h^−1^ DM, while in broad bean seeds L-DOPA yields can vary from 3.4 to 46 kg ha^−1^ [4,13], depending on the variety.

*V. faba,* besides containing L-DOPA [14], can have other phenolic compounds. For example, isoflavones such as daidzein and genistein are found in seedlings 15–20 DAS [15] and in the stem of plants 30–40 DAS [16], and in sprouts [17] there are kaempferol glucosides. These compounds and others found in the vegetative structures of the plant could have a beneficial biological effect on health. For this reason, studies are needed to identify what other phytochemicals with bioactive properties can be found in this species.

It has also been reported that *V. faba* can have antioxidant and anti-inflammatory effects. For example, the faba bean seed coat has antioxidant and anti-inflammatory activity [18], and in plants 10–15 days after emergence (DAE) only antioxidant activity has been reported [14], but it is not known whether they have an effect as an anti-inflammatory.

The hypothesis of this study was that the leaves and stem of Mexican varieties of broad beans have different contents and yields of L-DOPA and other bioactive compounds that have antioxidant and anti-inflammatory effects. Thus, the objective was to determine in plants 20 DAE the content and yield of total phenolic compounds, total flavonoids, and L-DOPA of four Mexican broad bean varieties and to analyze the flavonoid profile in the plant extract and the antioxidant and anti-inflammatory activity in vitro.

The results of this study show that young *V. faba* plants had high contents of TPC, TF, L-DOPA, rutin, and isoorientins and can be cultivated for use as a medicinal plant, a functional food, a nutraceutical, or for production of a natural drug for health improvement, mainly for PD patients.

## 2. Results and Discussion

### 2.1. Bioactive Compounds

The analysis of variance of the results for TPC, TF, L-DOPA, DPPH (IC_50_), and AANTI (IC_50_) of the varieties of the broad bean plants showed a significant effect (*p* ≤ 0.05 and *p* ≤ 0.01) in all the variables, except AANTI (IC_50_).

#### 2.1.1. Total Phenolic Compounds and Total Flavonoids

The TPC contents of the four broad bean varieties fluctuated between 111.82 and 135.63 mg GAE g^−1^ DM. The highest levels, though statistically equal (*p* ≤ 0.05), were detected in the varieties Matlazinca (135.63 mg GAE g^−1^ DM) and Rojita (131.65 mg GAE g^−1^ DM) (Table 1). These values are higher than those reported by Ortiz et al. [14] (94.16 mg GAE g^−1^ DM). It seems that the TPC content in the plant is higher than that in broad bean seed analyzed in other studies. For example, in major and minor botanical varieties, contents of 2.62 and 4.3 mg GAE g^−1^ DM [19] and 30.93 and 42.44 mg of catechin equivalent g^−1^ DM [18], respectively, have been reported. These values are lower than those found in our study for plants 20 DAE.

TF content varied from 91.98 to 118.58 mg QE g^−1^ DM. The highest values, though statistically equal (*p* ≤ 0.05), were found in the varieties Matlazinca and Rojita (118.58 and 111.17 mg QE g^−1^ DM, respectively) (Table 1). The plant TF content was higher than that found in another study with intact broad bean seed (testa + cotyledon) of the major (0.11 mg g^−1^ QE DM) and minor (0.14 mg g^−1^ QE DM) varieties [18].

TPC and TF contents lower than those found in our study can be found even in plants of other species considered medicinal. For example, the flowers of different wild medicinal species, such as *Crataegus monogyna*, *Cytisus multiflorus*, *Malva sylvestris*, and *Sambucus nigra*, also have TPC contents that vary from 5 to 55 mg g^−1^ DM [20]. Also, medicinal plants recognized in India had lower TPC and TF contents, such as in *Aerva lanata* (0.227 mg GAE g^−1^ DM and 0.069 mg QE g^−1^ DM), *Hygrophila schulli* (0.075 mg GAE g^−1^ DM and 0.003 mg QE g^−1^ DM), and *Biophytum reinwardtii* (0.135 mg GAE g^−1^ DM and 0.018 mg QE g^−1^ DM) [21].

#### 2.1.2. L-DOPA

The variety Rojita had the highest content of L-DOPA (88.04 mg g^−1^ DM), but it was not significantly different (*p* ≤ 0.05) from the varieties ICAMEX-V31 and Matlazinca (Table 1). Etemadi et al. [5] reported that the highest content of L-DOPA in *Vicia faba* organs (root, seedling, stem, leaf, flowers, pod, seed) was extracted from seedlings 15 days after germination [13.3 mg g^−^¹ fresh matter (FM)]. However, considering the water content (~80%) in fresh plant tissue [22], the values obtained in our study are comparable and even higher in some varieties such as Rojita, ICAMEX-V31, and Matlazinca than those reported by Etemadi et al. [5] (Table 1).

#### 2.1.3. In Vitro Antioxidant and Anti-Inflammatory Activity

**Figure 1:**
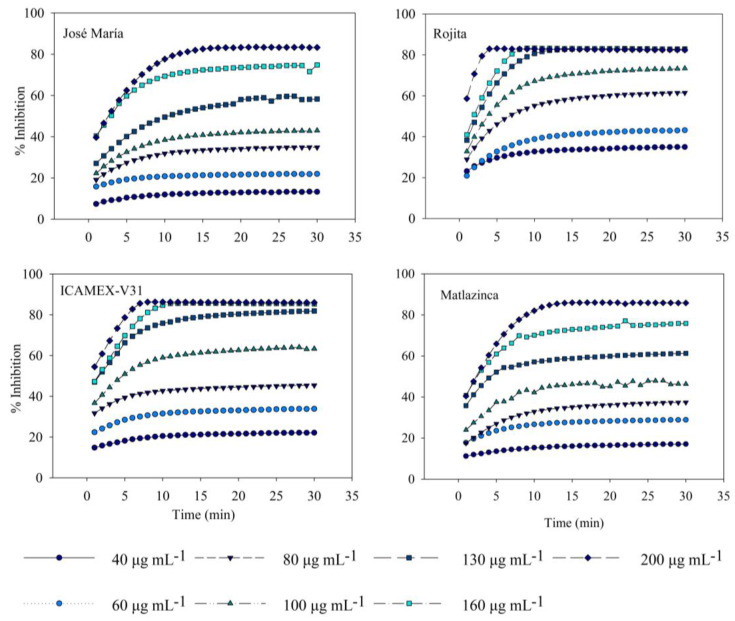
Antioxidant activity reported as inhibition percentage (I%) of the plant extracts from *Vicia faba* L. varieties 20 days after emergence.

Extracts from plants showed strong radical capture activity; the concentration of 200 μg mL^−1^ presented the highest percentage of inhibition, around 80% at 10 min of the reaction (Figure 1). The variety Rojita had the highest antioxidant activity (IC_50_ = 87.68 µg mL^−^¹), which was significantly different (*p* ≤ 0.05) from the varieties José María and Matlazinca (Table 1). Ortiz et al. [14] reported antioxidant activity in plant extracts of broad bean varieties 20 DAE that were lower than those reported in our study (Diamante IC_50_ = 121.04 µg mL^−1^ and Zac-22 IC_50_ = 130.27 µg mL^−1^). The antioxidant activity of the broad bean plant extract can be attributed to the high content of L-DOPA; a study [17] revealed that in extracts from broad bean sprouts this metabolite contributed strongly to antioxidant activity, unlike kaempferol glucosides. *V. faba* sprouts have been reported to have a greater antioxidant effect than the fruit or sprouts of other species. Okumura et al. [17] showed that broad bean sprouts have a higher percentage of DPPH radical capture activity at 100 μg mL^−1^ (approximately 70%) than the fruit (green broad beans) that have approximately 10% and higher than white radish, broccoli, and red cabbage sprouts (approximately 15, 12, and 25%, respectively). For this reason, the extract from the broad bean plant could be a good source of antioxidants. However, certain important aspects should be considered since it has been demonstrated that the antioxidant activity of *V. faba* fruit depends on factors such as the stage of seed maturity, the tissue or the fraction that is being evaluated, as well as the cooking methods [23]. Therein lies the importance of our study, which obtained the samples of four broad bean varieties to be compared from plants strictly 20 DAE.

**Figure 2:**
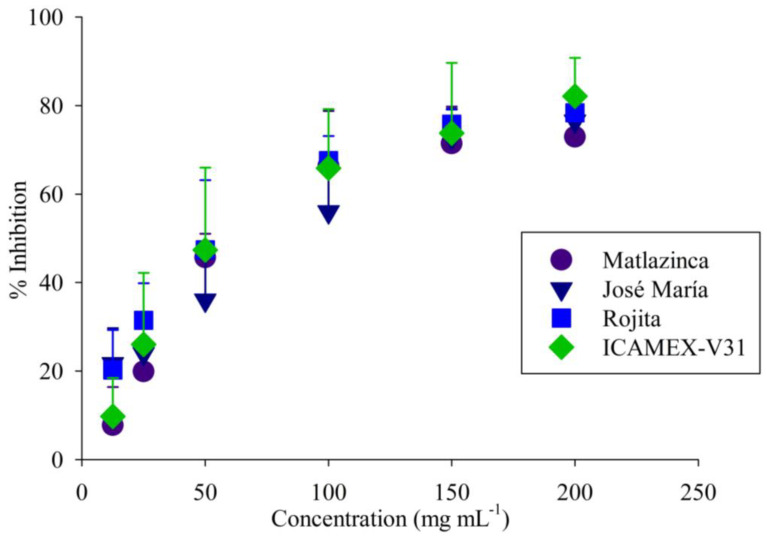
Anti-inflammatory activity of extracts from varieties of broad bean plants. Inhibition percentage from 12.5 to 200 mg mL^−1^. Each symbol represents the mean value of four independent replicates, and the error bar shows the standard error.

Regarding anti-inflammatory activity, the percentage of inhibition had a similar trend in all the extracts (Figure 2). At a concentration of 150 mg mL^−1^, the variety Rojita had the highest inhibition percentage (76%). The IC_50_ of the extracts of all the varieties was on average 87.90 mg mL^−1^; the variety Rojita showed the highest anti-inflammatory activity with a IC_50_ of 74.40 mg mL^−1^, which was not significantly different (*p* ≥ 0.05) from the other varieties (Table 1).

Diclofenac sodium is an anti-inflammatory drug; its main mechanism of action is related to inhibition of prostaglandin synthesis by reversible inactivation of the enzyme cyclooxygenase [24]. This drug was used as reference in the assay to detect inhibition of the turbidity caused by denaturation of the albumin protein from bovine serum induced by heat. The IC_50_ of the diclofenac sodium (at a concentration of 25 to 1000 μg mL^−1^) was 204.36 μg mL^−1^, which was higher than that of the plant extracts from the broad bean varieties Rojita (IC_50_ = 74.40 mg mL^−1^), ICAMEX-V31 (IC_50_ = 99.14 mg mL^−1^), José María (IC_50_ = 90.22 mg mL^−1^), or Matlazinca (IC_50_ = 87.87 mg mL^−1^), but our results demonstrate for the first time that the tissue of the broad bean plant has an in vitro anti-inflammatory effect against protein denaturation. This effect may be due to the type of polyphenols, mainly flavonoids, that are synthesized in the plants at this age (20 DAE). The effect inhibiting denaturation of the albumin of bovine serum has also been demonstrated with other species, such as with the extract of the plant *Oxalis corniculata*, with inhibition of 85.92% at a concentration of 0.8 mg mL^−1^ (IC_50_ of 0.288 mg mL^−1^) [25], and with the extract from *Syzygium zeylanicum* leaves at a concentration of 0.25 mg mL^−1^, which showed inhibition of 76.92% [26].

*V. faba* has also been reported to have an anti-inflammatory effect in vitro, but with the inhibitors of lipoxygenase (15-LOX) and extracts of faba bean seed coat at a concentration of 114.1 µg mL^−1^ of the variety *V. faba* minor and 192.2 µg mL^−1^ of the variety *V. faba* major showed an inhibition of 50% of the enzyme LOX [18].

### 2.2. Bioactive Compounds Determined by HPLC: Concentration of L-DOPA, Rutin, and Isoorientins

The contents of L-DOPA, rutin, and isoorientins in the methanol extracts were 512.01 g kg^−1^, 5.32 g kg^−1^, and 41.39 g kg^−1^, respectively. There were significant differences (*p* ≤ 0.05) among the broad bean varieties in L-DOPA and rutin contents.

**Figure 3:**
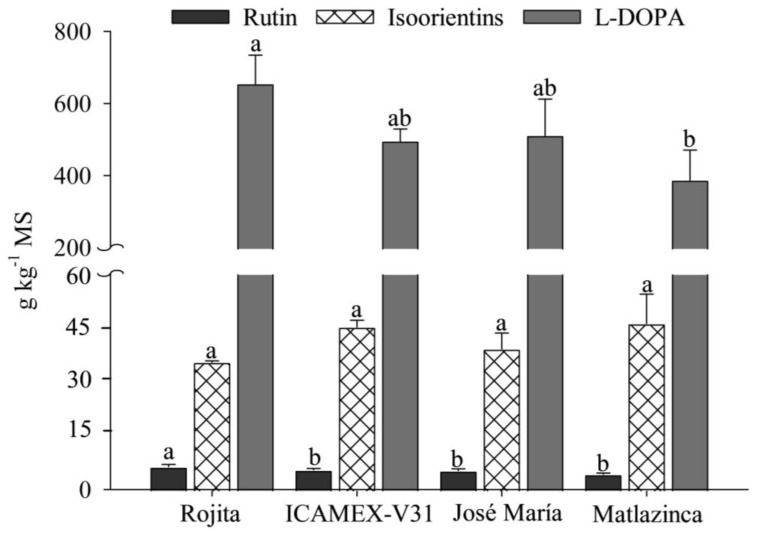
Contents of rutin, isoorientins, and L-DOPA in plants of four varieties of *Vicia faba* L. Bars with different letters within each compound indicate significant differences at *p* ≤ 0.05 according to Tukey’s test.

The variety Rojita had the highest accumulation of the metabolites L-DOPA, rutin, and isoorientins, about 700 g kg^−1^. Moreover, it had the highest content of rutin and L-DOPA, and, although the content of isoorientins was not the highest (35.34 g kg^−1^), it was not significantly different from that of the other varieties (*p* ≤ 0.05). The variety Matlazinca had the lowest accumulation of the analyzed metabolites, but it had the highest concentration of isoorientins (46.07 g kg^−1^) (Figure 3).

The compound rutin is a bioflavonoid, to which different effects are attributed: antioxidant, anti-inflammatory, neuroprotector, nephroprotector, hepaprotector, and anti-hyperglycemic. The anti-hyperglycemic effect against complications from diabetes includes a decrease in absorption of carbohydrates in the small intestine and inhibition of tissular gluconeogenesis, among others, which are well documented by Ghorbani et al. [27]. Rutin is an antioxidant compound that has a wide range of pharmacological applications. Moreover, it has been suggested that it can be used for treatment of chronic diseases such as cancer, diabetes, hypertension, and hypercholesterolemia. Compared with other flavonoids, rutin is a non-toxic, non-oxidable molecule [28]. However, one disadvantage is its low viability caused by its low water-solubility and instability, limited permeability, and low lipo-solubility that limit its use in topical applications [29].

In our study, the rutin content found in the extracts was 4.26–6.23 mg g^−1^, and the variety Rojita had the highest content. Gullón et al. [29] documented the rutin yield in a series of species that have different contents, among which *Valeriana officinalis* L. has 1.6 μg g^−1^, *Amaranthus paniculatus* 14.3 mg g^−1^, and *Flos Sophorae Immaturus* 251.4 mg g^−1^. This metabolite has anti-inflammatory activity, which has been evaluated in different trials in vitro and in vivo [29]. For example, Hao et al. [30], in rat models, found that rutin has an inhibitory effect on neuroinflammation as well as a neuroprotector effect and that this metabolite can improve healing of lesions such as edema, destruction of the hematoencephalic barrier, neurological deficit, and neuron death.

Isoorientins are a C-glucosyl flavone to which are attributed antioxidant, anti-inflammatory, antidiabetic, and antiobesity properties, and it can attenuate metabolic complications. The literature documents the presence of isoorientins in diverse plant species, mainly in those that have strong antioxidant properties. Examples include *Aspalathus linearis*, *Sasa borealis*, *Patrinia villosa*, and *Scabiosa stellata*, which can have isoorientins contents of 0.01–8.04 g 100 g^−1^ [31]. In our study, the extracts had contents of 3.5–4.6 g 100 g^−1^, suggesting that the broad bean plant could be an important source of this metabolite. Isoorientins have been shown to have potent anti-inflammatory properties both in vitro in rat macrophagous cell lines and in vivo reducing edema in the paw in rat models [32].

The in vitro assay on anti-inflammatory activity showed that the extract from the broad bean plant had an anti-inflammatory effect comparable to diclofenac sodium. This anti-inflammatory effect could be attributed to these metabolites, rutin and isoorientins, which are present in the broad bean plant.

L-DOPA is a non-protein amino acid with the structure of a phenolic acid [33]. This compound, used in the treatment of Parkinson’s disease, can easily cross the hematoencephalic barrier so that dopaminergic neurons can synthesize dopamine through the action of the enzyme L-dopa decarboxylase [34], which is the first of the three existing neurotransmitters of catecholamine (dopamine, noradrenaline, and adrenaline) [35]. When L-DOPA is administered orally, before reaching the brain, it can be catalyzed by dopa decarboxylase, monoamine oxidase, and catechol O-methyltransferase, which can cause vomiting and nausea, among other affectations. For this reason, the administration of L-DOPA together with inhibitors of peripheral decarboxylase enzymes can improve the bioavailability of L-DOPA [34]. In this way, L-DOPA restores the dopamine lost to degeneration and neuron death, reactivating motor functions and reducing disease symptoms.

Although this study did not consider evaluating the enantiomeric purity of L-DOPA, it is known that, in most cases, chiral natural products occur in nature in optically pure form, where only one enantiomer is biosynthesized in the producing organism [36]. For example, *Mucuna pruriens*, which is also a legume, is characterized as a major source of L-DOPA. In a study of dietary supplements containing *M. pruriens*, DOPA was enantiomerically analyzed; in all samples (14 samples), only L-DOPA was detected and in no sample was D-DOPA identified [37]. This suggests that in *Vicia faba* L. something similar could occur, but this still needs to be tested.

The plant extract could be a substitute for synthetic L-DOPA. The natural source could have fewer of the secondary effects caused by the drug, and it may help to delay progression of the disease [8]. L-DOPA contents were 387–654 g kg^−1^, depending on the variety. These quantities are highly promising and should be evaluated in vitro or in an in vivo model to confirm its effectiveness against Parkinson’s disease. Moreover, the compound has antioxidant activity, making it possible to be used in other types of pharmacological applications. It should be highlighted that these bioactive compounds found in the *V. faba* plant, L-DOPA, rutin, and isoorientins, may have a synergic effect that could prevent disease or serve as treatment of diseases such as Parkinson’s disease. Currently, Parkinson’s disease treatments have no neuroprotective activity [38], but considering that rutin and isoorientins have an anti-inflammatory effect, there is the possibility that, besides having a symptomatic effect [6], the broad bean plant could also have a neuroprotector effect on patients.

### 2.3. Yield of Bioactive Compounds

The biomass determined in fresh plant material varied from 10.58 to 16.28 g per plant. The variety José María had the highest biomass content, while the variety Matlazinca had the lowest biomass content. The variety José María also produced the highest quantity of biomass per unit of area (639.59 g m^−2^ FM), which was significantly different (*p* ≤ 0.05) from the others (Table 2).

The biomass determined in dry matter showed a similar trend. The variety José María also had the highest content of biomass both per plant (2.11 g) and per unit of area (83.25 g m^−2^ DM), which was significantly different (*p* ≤ 0.05) from the rest (Table 2).

Because yield is the product of the concentration of the metabolite and its biomass [4,5,39], the estimated yield of phenolic compounds, flavonoids, and L-DOPA per g plant^−1^, g kg^−1^, and g m^−2^ varied among the broad bean varieties. The highest yields per plant of TPC, TF, and L-DOPA were found in the variety Rojita (0.25 g plant^−1^ DM, 0.21 g plant^−1^ DM, and 0.17 g plant^−1^ DM, respectively). Per kilogram, the variety Rojita also had the highest contents of TPC (131.65 g kg^−1^ DM) and L-DOPA (87.53 g kg^−1^ DM) that were significantly different (*p* ≤ 0.05) from the other varieties, while the TF content of the Matlazinca variety was higher (113–56 g kg^−1^ DM) and significantly different (*p* ≤ 0.05) from the other varieties. Per unit of area, the variety José María had the highest content of TPC, TF, and L-DOPA (9.30 g m^−2^ DM, 8.08 g m^−2^ DM, and 5.64 g m^−2^ DM, respectively). However, these values were not significantly different from those of Rojita or from TPC and L-DOPA contents of ICAMEX-V31 (Table 2).

These results show that both the contents of metabolites and biomass are parameters necessary to evaluate yields of bioactive compounds. In this way, varieties can be selected for particular attributes of interest. Yields of the bioactive compounds present in the broad bean plant are abundant. Therefore, intensive production of plants of these varieties can be considered for consumption, use, or transformation into natural supplements for the prevention or treatment of diseases.

### 2.4. The Broad Bean Plant as a Source of L-DOPA for Parkinson’s Disease Patients

In this study, Mexican varieties of broad bean plants were shown to have high contents of phenolic compounds, L-DOPA, and flavonoids, as well as rutin and isoorientins. Thus, Parkinson’s disease patients could consume the plant and have a more complete, or possibly better, natural treatment than with the corresponding drug. First, it supplies L-DOPA, which is the main precursor for the brain’s production of dopamine and improves motor function [34,35]. Second, it contains flavonoids that have a neuroprotective effect. In this respect, Magalingam et al. [40] refer to the literature on the diverse mechanisms of flavonoids as neuroprotectors that can help to delay loss of neurons during progression of Parkinson’s disease. Thus, the broad bean plant could contribute both symptomatic effects and neuroprotective effects that are essential for Parkinson’s disease patients.

In Mexico, the Instituto Nacional de las Personas Adultas Mayores [41] reports that Parkinson’s disease is the second most common neurodegenerative disorder among adults 50 years or older. Although there are no exact figures, the Instituto Nacional de Neurología y Neurocirugía estimates 50 new cases per 100 thousand inhabitants per year. Worldwide, around five million people older than 50 years are victims of the disease.

Currently, L-DOPA continues to be the most prescribed drug for treatment of Parkinson’s, and *V. faba* is a natural source of L-DOPA and other bioactive compounds that can be used as an alternative in the place of chemically synthesized drugs. Administration of L-DOPA to Parkinson’s disease patients varies depending on how advanced the disease is, not on age. Apaydin et al. [42] and Cassani et al. [43] report the daily doses of synthetic L-DOPA usually taken daily by patients (Table 3).

The L-DOPA yields we obtained in plants were within a range of 110–170 mg plant^−1^ (Table 2). As a reference, these results suggest that a patient with Parkinson’s administered a dose of 300 mg L-DOPA a day as treatment (Table 3) could consume 2 to 3 dry broad bean plants harvested 20 DAE during the day (depending on the variety Rojita, ICAMEX, José María, or Matlazinca), which would be the equivalent of 300 mg of L-DOPA. Based on biomass, two plants would be equivalent to 2.92–4.22 g DM; three plants would be equivalent to about 4.38–6.33 g DM, depending on the variety (Table 2). It has been demonstrated that consumption of L-DOPA from broad bean seedlings (40 g fresh seedlings) increases the levels of L-DOPA in the plasm of Parkinson’s disease patients, who express improvement in motor characteristics similar to those obtained with the synthetic drug [6]. Thus, based on our results and the knowledge that the L-DOPA content in broad bean plants can be important for the control of Parkinson’s, we propose *V. faba* as a medicinal plant, through direct consumption of the plant or as raw material for production of a functional product, supplement, or nutraceutical. However, we emphasize that, prior to its use and consumption, it is necessary to conduct studies in vitro or in vivo with models to demonstrate and evaluate its symptomatic effect as well as its possible neuroprotective effect.

According to the review conducted by Pugalenthi and Vadivel [44], among the plants that contain L-DOPA, the *M. pruriens* plant has a sufficient quantity of the compound to be used as medicine. The seeds have the highest content (around 4.93–5.39%), while other organs of the *M*. *pruriens* plant have low contents: 0.17–0.35% in leaves, 0.19–0.31% in stems, and 0.12–0.16% in roots. Cassani et al. [43] demonstrated that consumption of *M. pruriens* seeds by Parkinson’s disease patients generated improvement in motor symptoms as well as longer duration in ON (active) and reduced dyskinesias. However, despite its effectiveness as a natural compound for Parkinson’s patients, obtaining *M. pruriens* seed takes between eight and nine months [45]. This contrasts with the young broad bean plant that can be harvested in about a month and a half after sowing. Moreover, *M. pruriens* is a seed that is not commonly consumed because of the anti-nutritional compounds it contains, including, among others, oligosaccharides (verbascose, stachyose, raffinose) [46] that can cause flatulence, interfere in absorption of nutrients in the intestine [47], stomach inflammation, and discomfort [48]. However, although anti-nutrients like oligosaccharides can be reduced with certain processing methods, such as soaking in a solution of NaHCO_3_ (0.2%) and cooking in an autoclave at 121 °C for 30 min, these processes can reduce the L-DOPA contents by 69–83% [49]. Thus, *V. faba* plants are a natural alternative for obtaining L-DOPA since they have contents of 6.78–8.75% (depending on the variety), similar to those accumulated in different varieties of dry *M. pruriens* seeds (1.21–9.49%) [43]. Moreover, in broad bean plants, there are apparently no oligosaccharides, and no patient has commented that consuming fresh broad bean plants caused flatulence or other discomfort [6].

*V. faba* is cultivated in Mexico and in several other countries such as Canada, as well as a large part of South America, Asia, Europe, Oceania, and part of Africa [50]. Thus, this species could supply L-DOPA to Parkinson’s patients, mainly to those with limited resources who cannot easily obtain the drug and who live in places where the legume can be grown. This is a proposal similar to that of Cassani et al. [43] through the seeds of *M. pruriens.*

Among the advantages of the broad bean plant, in contrast with *M. pruriens* seed, is the short period required from sowing to harvest, approximately 40 days, and the agronomic practices required are minimal. Moreover, it can be cultivated all year, and because of the short period needed for plant development, environmental pollution is minimal or nil. The possibility of obtaining L-DOPA with no oligosaccharide content from broad bean plants in a short period is highly advantageous, relative to *M. pruriens*, for use as a source of this phytochemical. Moreover, in our study, we demonstrated that these plants also contain phenolic compounds, flavonoids, rutin, and isoorientins, which could enhance the beneficial effect of L-DOPA in the treatment of Parkinson’s disease. This could lead to the beginning of a strategy to use *V. faba* to obtain bioactive compounds as an option for agricultural development. Also, the use of *V. faba* with this functional aim is a clear example of paradigm change, since crop production should not focus only on vegetables, fruits or legumes for nutritional food (proteins, carbohydrates, fats, vitamins, minerals), but also on production of bioactive compounds so that the consumer may have nutritive, healthy food that can contribute to minimizing disease.

## 3. Materials and Methods

### 3.1. Plant Material

The varieties of Mexican broad beans Rojita, ICAMEX V-31, José María, and Matlazinca were provided by the Instituto de Investigación y Capacitación Agropecuaria Acuícola y Forestal del Estado de México (ICAMEX) (19°14′26″ N 99°35′14″ W). The broad beans were sown in the field in the community of San Agustín Calvario de San Pedro Cholula, Puebla (19°03′03″ N, 98°20′37″ W) on 11 September 2019, at a depth of 5 cm, 2 seeds per hole, with 20 cm between holes in 80 × 60 cm plots. Plant density was higher than the usual density sown by farmers in the area (85 cm between rows and 70 cm between plants). Each plot was a replication, and four replications of each variety were established.

### 3.2. Identification, Collection, and Processing of the Plant Material

Every day, between 8:00 and 10:00 h, we inspected plant emergence and identified each plant with colored yarn to monitor their development. Plants of the same variety were harvested 20 DAE (±1 day), and three groups per plot were formed of three plants without roots. The plants were chopped, placed in a paper bag, and weighed on an analytical balance. They were then dried in a forced convection oven (SHEL LAB, 1370FX, Cornelius, OR, USA) at 38 °C for 36 h approximately until the percentage of moisture in the plant material was 7 to 10%, determined in a thermobalance (OHAUS, MB 45, Washington, DC, USA). The dried plant material was pulverized in a coffee grinder (KRUPS, GX4100, USA), sifted to 500 μm particle size, and stored refrigerated in cellophane bags at 4 ± 1 °C for later use in the different determinations. For each broad bean variety, we recorded the quantity of plants obtained per plot to evaluate yield of the bioactive compounds per plant, per kilogram, and per unit of area (m^2^).

### 3.3. Agri-Environmental Variables

**Figure 4:**
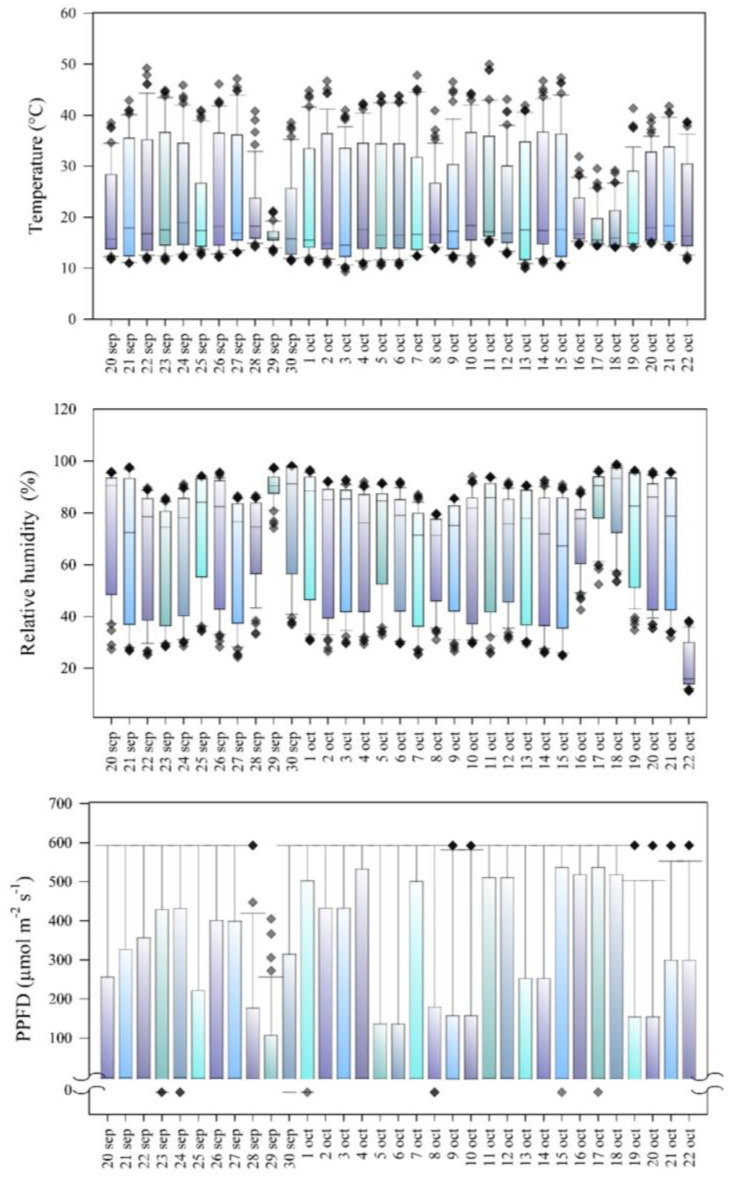
Agri-environmental variables during *Vicia faba* plant development 20 days after emergence. PPFD: photosynthetic photon flux density. Each box represents 48 data per day. The first and third quartiles show the largest distribution of values, and the second quartile shows the mean of the values. The arms represent the minima and maxima, and the extreme points represent the outliers.

In the experimental field, we recorded every 30 min the data of temperature (°C), relative humidity (%), and luminous intensity (expressed in photosynthetic photon flux density, PPFD, µmol m^−2^ s^−1^) with a datalogger (HOBO, U12–012, Washington, DC, USA). Figure 4 shows the agri-environmental variables during the period of plant development.

### 3.4. Analysis of Total Phenolic Compounds and Total Flavonoids

Total phenolic compounds and total flavonoids were extracted and analyzed following Herald et al. [51] with the modifications of Fuentes-Herrera et al. [3].

### 3.5. L-DOPA Analysis by Visible UV Spectrophotometry

Extraction. L-DOPA was extracted following the methodology proposed by Polanowska et al. [52] with a few modifications. Extractor solution (7 mL) of water acidulated with 0.2% (*v*/*v*) acetic acid was added to a 40 mg sample. The mixture was homogenized at 21,500 rpm for 20 s in an Ultra-Turrax (IKA, T25 basic S1, Wilmington, NC, USA). The tubes were then placed inclined in an orbital shaker (Daigger, OR100, Hamilton, NJ, USA) for 20 min. After 10 min, the tubes were spun and left to rest during the rest of the time. The mixture was vacuum filtered through No. 42 filter paper (Whatman, 1442-055), and the extract was placed in Eppendorf tubes for analysis.

**Figure 5:**
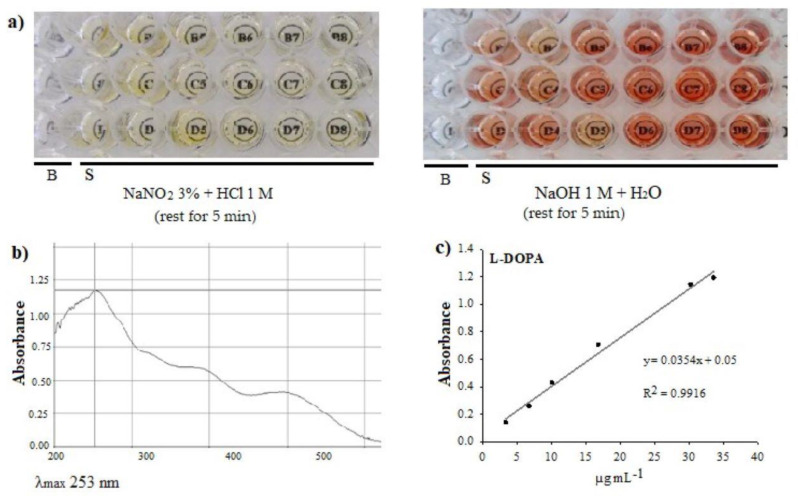
Determination of L-DOPA by visible UV spectrophotometry in microplate, (**a**) reagents and color of the reactions to determine L-DOPA content; B: blank, S: sample, (**b**) absorbance spectrum, 200–600 nm, (**c**) calibration curve.

Analysis. The methodology proposed by Rahmani-Nezhad et al. [53] was adapted to carry out the analysis in microplates. We placed 10 µL of the extract (5.7 mg mL^−1^) or a standard in a flat-bottom UV microplate and added 20 µL of 3% (p/v) NaNO_2_ and 10 µL HCl at 1 M. The mixture was shaken and left in repose for 5 min; this turned yellow. After adding 30 µL NaOH 1 M and leaving it to rest for 5 min, the mixture turned a reddish color. After this time, 180 µL distilled water was added and the mixture shaken, and absorbance was recorded at 253 nm in a visible UV spectrophotometer (Thermo Scientific, Varioskan flash, Vantaa, Finland) (Figure 5). To define the wavelength, a scan in the range of 200–600 nm against a blank (containing the same reagents, except for the sample) was previously performed on the extract, and the maximum absorbance was recorded at a λ of 253 nm. The L-DOPA standard (Sigma, No. Cat. PHR1271) was dissolved with a solution of 0.2% acetic acid, obtaining a concentration of 84 µg mL^−1^, which was used to construct the calibration curve of 3.36 to 33.6 µg mL^−1^.

### 3.6. Antioxidant Activity

Extraction. A 5 mg sample of dehydrated plant tissue was added to 5 mL methanol. The mixture was shaken for 5 s in vortex and extracted with ultrasound for 30 min (Ultrasonic Cleaner, AS5150B, Wenzhou, China). The mixture was centrifuged at 7000 rpm for 5 min at 4 °C (HERMLE Labortechnick, Z 326 K, Wehingen, Germany). The supernatant was placed in Eppendorf tubes to be stored frozen at −20 °C until analysis.

Analysis. We used the 2,2-diphenyl-1-picrylhydrazyl (DPPH) methodology based on Brand-Williams et al. [54] and Rahman et al. [55], with modifications. The reagent DPPH (Sigma-Aldrich, D9132) was mixed with methanol to a concentration of 0.1 mM. In a Costar microplate (96 flat-bottomed wells), we placed 50 µL of the extract at different concentrations (40, 60, 80, 100, 130, 160, and 200 µg mL^−1^) and added 200 µL DPPH at 0.1 mM. The plate was placed in the spectrophotometer (Thermo Scientific, Varioskan flash, Vantaa, Finland) and shaken for 30 s. Absorbance was recorded every minute at 521 nm for 30 min (at room temperature in the dark). As the blank, we used 50 µL methanol in the place of the extract. With the results, we obtained the inhibition percentage (I%), which corresponds to the quantity of the DPPH radical reduced by the extract at a given concentration (Equation (1)). We also calculated the inhibitory concentration (IC_50_), which is the concentration of the sample required to eliminate 50% of the DPPH free radicals [56]. The IC_50_ value was obtained by graphing the concentration against the result of I% at minute 30. Through linear regression, we obtained the equation of the line from which X was cleared. The Y value was 50, representing 50% inhibition. In this way, the X value represented the concentration of the sample that would achieve 50% inhibition of DPPH. Thus, the higher the antioxidant activity, the lower the value of IC_50_.

(1) $$I\%=\left[\frac{AB-AS}{AB}\right]\times 100$$

where

*I*%: Inhibition percentage;
*AB*: Absorbance of the blank;
*AS*: Absorbance of the sample.

### 3.7. In Vitro Assay of Anti-Inflammatory Activity

Sample extraction. We added 100 mL methanol (MERCK, MX0485-7) to 2.5 g dehydrated plant tissue. The mixture was sonicated twice for 10 min at power 5 and vacuum filtered through No. 42 filter paper (Whatman, 1442-055). The filtrate was concentrated in a rotavapor (Heidolph, Laborota 4000, Schwabach, Germany) at 37 °C until dry. The residue was resuspended in 2 mL distilled water. Using this solution, different dilutions were prepared, from 12.5 to 200 mg mL^−1^.

**Figure 6:**
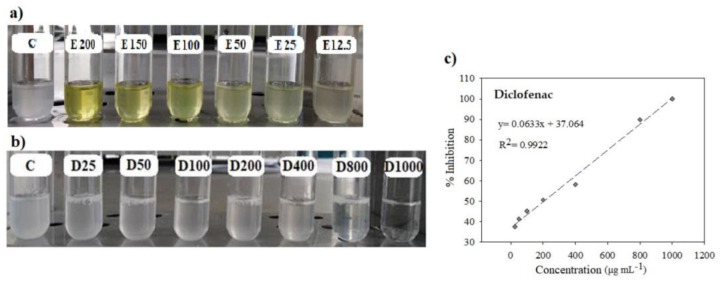
Anti-inflammatory activity assay. (**a**) Inhibition effect on protein turbidity of the broad bean plant tissue extract at different concentrations (12.5–200 mg mL^−1^); C: negative control, E: extract. (**b**) Inhibition effect on protein turbidity generated by the diclofenac sodium at different concentrations (25–1000 μg mL^−1^); C: negative control, D: diclofenac. (**c**) Inhibition percentage of diclofenac sodium.

Analysis. The analysis was conducted according to the methodologies proposed by Mizushima and Kobayashi [57] and Sakat et al. [25], with modifications. The basis of the assay is that proteins are denatured by the action of heat, and drugs with anti-inflammatory action are able to stabilize proteins [57]. In glass tubes, we placed 450 μL of 1% bovine serum albumin (Sigma-Aldrich, 05470) diluted in distilled water, and 50 μL of the different concentrations (12.5, 25, 50, 100, 150, and 200 mg mL^−1^) of the extract was then added to each tube. For the negative control, we used 50 μL deionized water. The mixture was shaken for 10 s in vortex (Scientific Industries, SI-A236, Bohemia, NY, USA) and immediately the tubes were placed in a water bath (Thermo Scientific, 2841) for 30 min at 37 °C. After this time, we slowly increased the temperature of the water bath to 58 °C (the process lasted 30–32 min). The tubes were then left to cool to room temperature and 2.5 mL phosphate buffered saline (PBS) at 0.1 M, pH 6.3, was added (Figure 6). Turbidity of the mixture (protein denatured by heat) was measured with a spectrophotometer (Thermo Electron Corporation, Evolution 300, Madison, WI, USA) at 660 nm against the PBS vehicle. We used a vial of diclofenac sodium (Pharmalife^®^, Atlanta, GA, USA) to observe the inhibition effect on denaturation of the bovine serum protein. The diclofenac sodium was diluted with NaCl isotonic solution (J.T. Baker, 3624-01) to obtain the concentrations of 25, 50, 100, 200, 400, 800, and 100 μg mL^−1^ to be evaluated (Figure 6). We calculated the percentage of inhibition with Equation (2). With the results, we performed a linear regression analysis to obtain the value of IC_50_ that corresponded to the value of the sample concentration necessary to inhibit protein denaturation by 50%.

(2) $$\%\;I=\left[\frac{ANC-AS}{\;ANC}\right]\times 100$$

where

*I*: Inhibition;
*ANC*: Absorbance of the negative control;
*AS*: Absorbance of the sample.

### 3.8. L-DOPA, Rutin, and Isoorientins by HPLC Analysis

Extraction. We added 100 mL methanol (MERCK, MX0485-7) to 3 g dehydrated plant tissue. The mixture was sonicated twice for 10 min at power 5, then vacuum filtered through No. 42 filter paper (Whatman, 1442-055). The filtrate was concentrated and dried in a rotavapor (Heidolph, Laborota 4000, Schwabach, Germany) at 37 °C. The dried residue was then weighed on an analytical balance and stored in an amber jar under refrigeration (4 °C) until its analysis.

**Figure 7:**
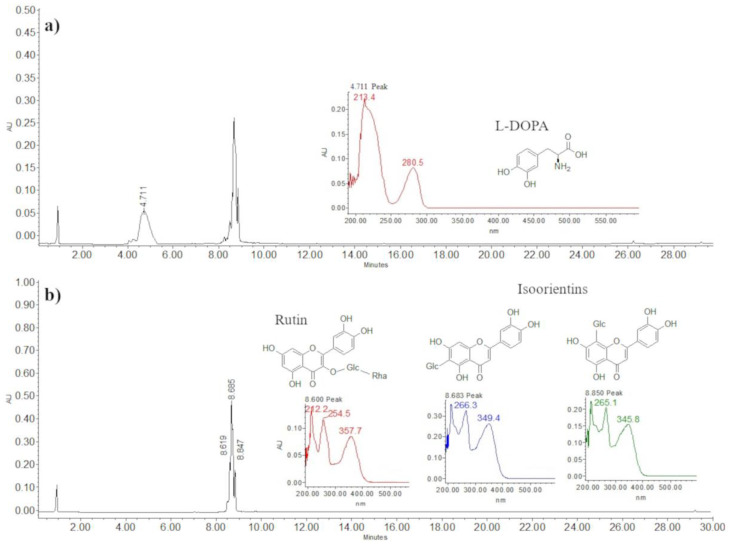
Chromatogram of the methanol extract of *Vicia faba* plant tissue:(**a**) L-DOPA, 280 nm; (**b**) rutin (peak 1) and isoorientins (peaks 2 and 3), 350 nm.

Analysis. Part of the residue of each extraction of the samples was diluted to a concentration of 2 mg mL^−1^ with 100% methanol. The dilution was filtered through 0.45 µm filter discs (CELLTREAT, 229744) and deposited in an amber vial. For the analysis, we used HPLC equipment (Waters, model Alliance 2695, Milford, MA, USA) with a diode array detector (Waters 2996) and a SUPELCO C18 column (25 mm long and 4.6 mm internal diameter, 5 µm particle size) (Sigma-Aldrich, 504971). HPLC-grade solvents were used for the mobile phases. Mobile phase A: acidulated water (water with 0.5% TFA: trifluoroacetic acid); mobile phase B: acetonitrile. We used a gradient during a run time of 30 min (Table 4). A total of 10 µL of sample was injected at a flow of 0.9 mL min^−1^. Temperature of the column was 27 °C, and pressure of the equipment was maintained at 1528 psi. Compounds were detected at 280 nm for L-DOPA and 350 nm for rutin and isoorientin. Compounds were quantified by comparing the retention times and the peak areas with standard curves for L-DOPA (31.25–1000 µg mL^−1^) (Sigma, PHR1271), rutin (12.5–200 µg mL^−1^) (Sigma, R5143), and isoorientins (12.5–200 µg mL^−1^) (Sigma, I1536) dissolved in 100% methanol. Retention times were the following: L-DOPA 4.71 min, rutin 8.6 min, and isoorientins 8.68 and 8.85 min (Figure 7).

### 3.9. Statistical Analysis

Under an experimental design of complete random blocks, we analyzed results for TPC, FT, L-DOPA, DPPH (IC_50_), and AANTI (IC_50_), obtaining the mean of four replications. The results for L-DOPA, rutin, and isoorientins were expressed as the mean of three replications. Biomass and yield of the bioactive compounds evaluated by plant, kilogram, and square meter were the product of the mean of three replications. The results were subjected to an analysis of variance and the Tukey comparison of means with a significance level of *p* ≤ 0.05, using SAS statistical software version 9 [58].

## 4. Conclusions

This study described the content and yield of L-DOPA and bioactive compounds, as well as the antioxidant and anti-inflammatory activity of broad bean plants of Mexican varieties (Rojita, ICAMEX-V31, José María and Matlazinca) 20 days after emergence. The plants showed significant contents of total phenolic compounds (TPC), total flavonoids (TF), and L-DOPA, as they showed higher values compared to other studies of plants of the same species or other species. The antioxidant activity of the faba bean varieties was higher than those detected in similarly aged plants of the same species, whereas the in vitro anti-inflammatory activity shown in the plant extract had not been documented in these vegetative structures. The flavonoid profile that revealed the presence of rutin and isoorientins had not been previously detected in *V. faba* plants. The antioxidant activity of the plants is attributed to the content of L-DOPA and phenolic compounds, while the anti-inflammatory activity could be associated with the presence of flavonoids, rutin, and isoorientins. The content of bioactive compounds and the antioxidant activity of the plant showed significant differences in relation to the broad bean variety, but the anti-inflammatory activity did not show significant differences between varieties. Remarkably, two varieties (José María and Rojita) produced the highest estimated yield per unit area and showed a high potential for accumulation of TPC, TF, and L-DOPA. These results showed that young *V. faba* plants have important contents of bioactive compounds, suggesting that they could be cultivated to be exploited as a medicinal plant, functional food, nutraceutical, or for the production of a natural drug to improve health, mainly for Parkinson’s patients. However, in vivo trials are needed to determine their efficacy.

## Figures and Tables

**Table 1:** Contents of bioactive compounds in varieties of *Vicia faba* L. plants 20 days after emergence.

Variety	TPC	TF	L-DOPA	DPPH (IC_50_)	AANTI (IC_50_)
	(mg GAE g^−1^ DM)	(mg QE g^−1^ DM)	(mg g^−1^ DM)	(µg mL^−1^)	(mg mL^−1^)
Rojita	131.65 a ± 15.44	111.17 a ± 9.97	88.04 a ± 10.02	87.68 a ± 2.49	74.40 a ± 13.27
Matlazinca	135.63 a ± 14.44	118.58 a ± 20.14	79.91 ab ± 5.46	97.42 c ± 2.49	87.87 a ± 10.55
ICAMEX-V31	127.03 ab ± 12.86	91.98 b ± 11.94	79.98 ab ± 10.55	94.79 ab ± 6.57	99.14 a ± 19.32
José María	111.82 b ± 7.26	92.76 b ± 10.84	67.30 b ± 18.55	106.55 d ± 5.21	90.22 a ± 16.47

TPC: total phenolic compounds, TF: total flavonoids, L-DOPA: L-3,4-dihydroxyphenylalanine, DPPH: antioxidant activity, AANTI: anti-inflammatory activity. Means in a column with different letters are statistically different, according to the Tukey test (*p* ≤ 0.05).

**Table 2:** Biomass and yield of bioactive compounds per plant, kilogram, and square meter of four varieties of *Vicia faba* L. plants.

	Rojita		ICAMEX-V31		José María		Matlazinca	
**Biomass** (g plant^−1^ FM)	15.24 a	±0.43	12.57 b	±0.24	16.28 a	±0.35	10.58 c	±0.81
**Fresh plants** (g m^−2^ FM)	471.82 b	±13.60	434.94 b	±53.24	639.59 a	±13.92	407.84 b	±51.05
**Biomass** (g plant^−1^ DM)	1.97 a	±0.03	1.73 b	±0.01	2.11 a	±0.03	1.46 c	±0.12
**Dry plants** (g m^−2^ DM)	61.12 b	±1.18	59.71 b	±5.97	83.25 a	±1.25	56.55 b	±8.24
**TPC**								
g plant^−1^	0.25 a	±0.00	0.21 b	±0.00	0.23 ab	±0.00	0.19 c	±0.01
g kg^−1^	131.65 a	±15.43	127.03 c	±13.35	111.77 d	±9.10	131.28 b	±12.80
g m^−2^	8.04 ab	±0.15	7.58 ab	±0.75	9.30 a	±0.14	7.42 b	±1.08
**TF**								
g plant^−1^	0.21 a	±0.00	0.16 c	±0.00	0.19 b	±0.00	0.16 c	±0.01
g kg^−1^	111.03 b	±11.00	92.44 c	±13.78	92.17 d	±13.79	113.57 a	±19.22
g m^−2^	6.78 ab	±0.13	5.52 b	±0.55	8.08 a	±0.12	6.42 b	±0.93
**L-DOPA**								
g plant^−1^	0.17 a	±0.00	0.13 b	±0.00	0.14 b	±0.00	0.11 c	±0.00
g kg^−1^	87.53 a	±11.11	80.48 b	±10.99	67.81 d	±18.94	79.68 c	±10.05
g m^−2^	5.34 ab	±0.10	4.80 ab	±0.48	5.64 a	±0.08	4.50 b	±0.65

TPC: total phenolic compounds, TF: total flavonoids, L-DOPA: L-3,4 dehydroxyphenylalanine. Data were analyzed in triplicate and results are presented as means ± standard deviation. Means in a row with different letters are statistically different, according to the Tukey test (*p* ≤ 0.05).

**Table 3:** L-DOPA dose requirements for patients with Parkinson’s disease.

	Patient						
	1	2	3	4	5	6	7
Gender	M	M	M	F	M	M	M
Age (years)	85	69	62	52	48	40	56
Dose of L-DOPA (mg d^−1^)	300	400	300	475	600	300	500

M: masculine, F: feminine. Authors’ construction with information documented by Apaydin et al. [42] and Cassani et al. [43].

**Table 4:** Gradient of mobile phases used in the analysis.

	Mobile Phase	
Time (min)	A (%)	B (%)
0	100	0
1	100	0
2	100	0
3	95	5
4	95	5
20	70	30
21	50	50
22	50	50
23	50	50
24	20	80
25	20	80
26	0	100
27	0	100
28	100	0
30	100	0

## Data Availability

All relevant data are already in the article.
